# Measurement Properties of the Modified Spinal Function Sort (M-SFS): Is It Reliable and Valid in Workers with Chronic Musculoskeletal Pain?

**DOI:** 10.1007/s10926-017-9717-y

**Published:** 2017-07-29

**Authors:** Maurizio Alen Trippolini, Svenja Janssen, Roger Hilfiker, Peter Oesch

**Affiliations:** 1 Department of Work Rehabilitation, Rehaklinik Bellikon, Suva Care, Bellikon, Switzerland; 2 Massachusetts General Hospital (MGH) Institute of Health Professions, PhD in Rehabilitation Sciences Program, Charlestown, Boston, MA USA; 3 School of Health Sciences, HES-SO Valais-Wallis, University of Applied Sciences and Arts Western Switzerland Valais, Sion, Switzerland; 4 Department of Research, Rehabilitation Centre Valens, Valens, Switzerland

**Keywords:** Back pain, Self-efficacy, Questionnaire, Validity, Work

## Abstract

*Purpose* To analyze the reliability and validity of a picture-based questionnaire, the Modified Spinal Function Sort (M-SFS). *Methods* Sixty-two injured workers with chronic musculoskeletal disorders (MSD) were recruited from two work rehabilitation centers. Internal consistency was assessed by Cronbach’s alpha. Construct validity was tested based on four a priori hypotheses. Structural validity was measured with principal component analysis (PCA). Test–retest reliability and agreement was evaluated using intraclass correlation coefficient (ICC) and measurement error with the limits of agreement (LoA). *Results* Total score of the M-SFS was 54.4 (SD 16.4) and 56.1 (16.4) for test and retest, respectively. Item distribution showed no ceiling effects. Cronbach’s alpha was 0.94 and 0.95 for test and retest, respectively. PCA showed the presence of four components explaining a total of 74% of the variance. Item communalities were >0.6 in 17 out of 20 items. ICC was 0.90, LoA was ±12.6/16.2 points. The correlations between the M-SFS were 0.89 with the original SFS, 0.49 with the Pain Disability Index, −0.37 and −0.33 with the Numeric Rating Scale for actual pain, −0.52 for selfreported disability due to chronic low back pain, and 0.50, 0.56–0.59 with three distinct lifting tests. No a priori defined hypothesis for construct validity was rejected. *Conclusions* The M-SFS allows reliable and valid assessment of perceived self-efficacy for work-related tasks and can be recommended for use in patients with chronic MSD. Further research should investigate the proposed M-SFS score of <56 for its predictive validity for non-return to work.

## Introduction

According to the Global Burden of Disease Study, musculoskeletal disorders (MSD) are a leading cause of work disability, inflicting substantial burden on society and the individual [1]. Long-term work disability in patients with MSD is often associated with low perceived self-efficacy [2]. According to Bandura, perceived self-efficacy (PSE) refers to the individual’s beliefs about their own competence and ability to undertake behaviors to achieve desired goals [3]. PSE affects how people behave in difficult situations, and people who doubt their capabilities shy away from tasks that they view as a personal threat. PSE is embedded in the theory of planned behavior (TPB). In the TPB it is assumed that intention to demonstrate a behavior is an important predictor for the actual behavior [4]. Behavioral intentions reflect the effort that people plan to behave in the valued direction and they are a function of: (a) the person attitude toward the behavior; (b) person’s perception of social norms regarding the behavior; and (c) the perceived behavioral control i.e. the person’s perception of ease or difficulty of the behavior [5]. By applying this model to rehabilitation it is assumed that patients with low levels of PSE are less likely to perform well on the tasks presented, and patients with high levels of PSE are assumed to perform better. In addition, PSE appears to be closely related to health outcomes [6] and return to work (RTW) in persons with MSD and psychological disorders [7, 8]. Therefore, measuring levels of PSE may be critical when tailoring interventions aimed to increase PSE and improve RTW outcomes.

An efficient way of assessing PSE in patients with MSD is the use of questionnaires. However, questionnaires have important limitations for their use in patients with diverse cultural backgrounds. The first is that the use of questionnaires depends on the literacy and linguistic skills of an evaluee, which may be limited in these patients [9]. The second is that only a few questionnaires assessing PSE have a work-related point of reference, but consider an unlimited spectrum of activities. These limitations may be overcome by using a picture-based questionnaire such as the Spinal Function Sort (SFS) [10]. The Spinal Function Sort (SFS) includes 50 depicted items, and claims to measure PSE to perform work-related tasks [10].

The SFS was shown to have good predictive value for work resumption in a multilingual European rehabilitation setting [11]. However, the rating of 50 items is time-consuming, and a shorter version of the SFS is warranted. It has also been suggested that the number of SFS items could be reduced by half because of redundant items [12, 13]. Another critique was that the SFS does not include pictures of prolonged work postures, ambulation and the images are outdated [12, 13]. Therefore, the 20-item Modified Spinal Function Sort (M-SFS) was developed using a mixed methods approach, which involved expert opinions, interviews with patients and item analysis based on data from clinical studies [14]. Validity and reliability of the M-SFS has not been established yet. The aim of this study is therefore assessing the validity, test–retest reliability and measurement error of the M-SFS in a group of patients with chronic nonspecific MSD in a rehabilitation setting.

## Methods

### Subjects, Context and Study Design

#### Subjects

This study was embedded within the usual care for patients with MSD referred for an interdisciplinary inpatient work rehabilitation due to the following reasons: (1) having plateaued with previous medical and rehabilitative care; (2) not regaining full work capacity (WC); (3) exceeding expected healing times. Patient recruitment took place from December 2015 to June 2016 in two different Swiss Rehabilitation centers, in Bellikon (Canton Argovie) and Valens (Canton St. Gallen). Inclusion criteria were: Patients with chronic (>3 months), nonspecific MSD, age between 18 and 65, willing to participate in the retest after 2 days and to sign informed consent. Patients were excluded if they were pregnant, reported acute comorbidities (cardio-pulmonary, psychiatric, neurologic or internal medical), had a medically determined limit in lifting capacity <25 kg or were insufficiently proficient in the German language to understand instructions or read the questions.

Ethical approval for this study was granted by the Medical Ethics Committee of the Cantons Aargau and St. Gallen (EK AG 2012/073).

#### Study Design and Procedure

A test–retest design was chosen to evaluate the measurement properties of the M-SFS. After checking for eligibility, patients filled out demographic data and various questionnaires, including the M-SFS described in the next paragraph. Retest was performed no earlier than 2 days later than the first test to reduce risk for recall bias. The functional capacity evaluation (FCE) tests were performed after completion of the M-SFS retest.

### Measurements

#### Modified Spinal Function Sort (M-SFS)

**Fig. 1:**
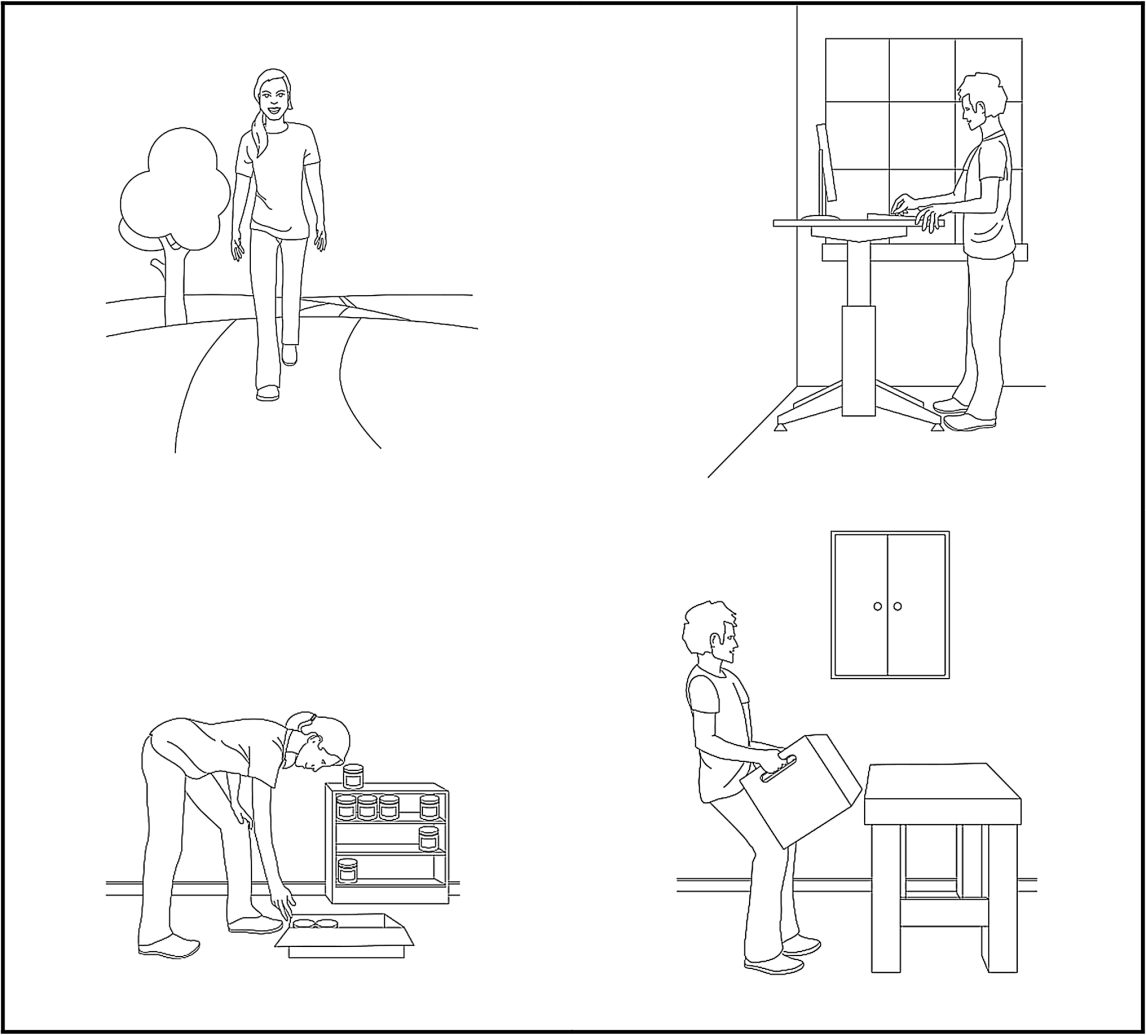
Drawings referring to items number 16, 3, 17, 18 (from *top left* to *bottom right*) of the Modified Spinal Function Sort (M-SFS) questionnaire

The M-SFS purports to measure PSE to perform work-related tasks. The M-SFS contains 20 drawings with simple written descriptions (see examples in Fig. 1). Patients rated their PSE for each task on a 5-point Likert scale ranging from “able” (4 points), to “restricted” (3, 2 or 1 points) or “unable” (0 points) adding up to a single rating score ranging from 0 to 80. Higher scores indicated higher PSE. The development of the M-SFS has been described in detail elsewhere [14].

#### Original Spinal Function Sort

The original Spinal Function Sort (SFS) measures PSE for work-related tasks that involve the spine [10]. The SFS contains 50 drawings with brief descriptions. The same 5-point Likert scale of the M-SFS is used in the SFS. The SFS yields a single rating ranging from 0 to 200, with higher scores indicating higher PSE. Validity and reliability of the SFS has been established for various musculoskeletal disorders, languages and settings [6, 7, 11, 12].

#### Pain Disability Index

The Pain Disability Index (PDI) is a self-report instrument assessing the degree to which chronic pain interferes with various daily activities [15]. The PDI contains seven categories of life activities: family, recreation, social activity, occupation, sexual behavior, self-care and life-support. A score of 0 means no disability at all, and a score of 10 signifies that all of the activities in which a person is normally involved are totally disrupted or prevented by pain. The total score ranges from 0 to 70, a higher score indicates more severe self-reported disability. Reliability and validity of the PDI as a brief measure of pain-related disability has been shown [16].

#### Oswestry Disability Index

Self-reported disability due to low back pain (LBP) was measured with the Oswestry Disability Index (ODI). The ODI contains 10 items: pain intensity, personal care, lifting, walking, sitting, standing, sleeping, sex life, social life and travelling. Each item includes five statements reflecting different levels of perceived disability ranging from no disability (0) to total disability (5). The total score ranges from 0 to 50, a higher score indicates more severe self-reported disability. The ODI is reliable and has been validated in several languages and settings [17].

#### Pain

Pain intensity was measured with an 11-point numeric rating scale (NRS) ranging from no pain (0) to worst pain (10). The patient was asked to rate their actual pain (“pain now”). The NRS is a commonly used scale with proven reliability and validity in patients with MSD with different levels of literacy [18].

#### Physical Performance

Functional capacity evaluation (FCE) is a standardized battery of functional tests that intend to measure a patient’s safe physical ability for work-related activity [19]. For the purpose of this study three lifting tests were analyzed: lifting floor to waist (“lifting low”), lifting waist to crown height (“lifting high”) and short two-handed carry (“horizontal lift”). Patients were asked to perform the test to their maximum ability. The tests are described in detail elsewhere [20]. In order to compare the results of this study with previous studies with the original version of the SFS we did include three FCE tests used in these studies only [11, 13]. That kept the burden of the study small for the participants. The three FCE tests have shown acceptable reliability and validity in patients with various musculoskeletal disorders and professional backgrounds [12, 17–20].

### Data Analysis

Normal distribution was visually assessed using P-P plots and tested with the Kolmogorov–Smirnov and the Shapiro–Wilk tests. Floor and ceiling effects for the M-SFS were considered to be present if more than 15% of participants achieved the lowest or highest possible score of the items [21].

#### Internal Consistency

Internal consistency was assessed by item-to-total correlations and Cronbach’s alpha. Optimal consistency for measurements at group level was considered when alpha value was between 0.7 and 0.9. Values <0.7 may be indicative for items measuring different traits, values >0.9 may be indicative for item redundancy [22].

#### Item-Structure Analysis

The dimensionality of the 20 M-SFS items was measured using principal component analysis (PCA) with Kaiser normalization and Varimax rotation. An Eigenvalue criterion of 1.0 was used for the analysis. Multidimensionality was assumed since the M-SFS contained new items about activities with tasks including postural tolerance and ambulation.

#### Test–Retest Reliability and Measurement Error

Test–retest reliability was expressed as an intraclass correlation coefficient (model 1; one-way random) (ICC). ICC was interpreted as follows: ICC ≥0.90 is excellent; good when ICC was between 0.75 and 0.90; moderate when ICC was between 0.50 and 0.75; and poor when ICC ≤0.50. ICCs were acceptable when ICC ≥0.75, and the lower boundary of the 95% confidence interval of the ICC ≥0.50 [23]. Measurement error was expressed in limits of agreement (LoA) (mean difference ± 1.96 × standard deviation of mean difference) [24]. The narrower the limits of agreement, the smaller the disagreement between the repeated tests.

#### Construct Validation: Hypothesis Testing

Four predefined hypotheses on the strength of the association between M-SFS and the original SFS, pain self-reported disability (ODI), self-reported pain (NRS) and physical performance (FCE tests) are displayed in Table 1. The strength of the association is expressed in the absolute value of the correlation coefficient. Associations were calculated using Spearman’s rank or Pearson’s correlation coefficient as appropriate and interpreted as follows: 0.00–0.29 are “small”; 0.30–0.49 are “medium”; and 0.50–0.99 are “large” in terms of the magnitude of the correlation [25]. The rational for the four hypotheses was the following: although new items were added, the M-SFS was expected to be highly correlated due to the overlap in 12 items between the two the original SFS and the M-SFS. We expected moderate correlations between the M-SFS and the two pain measures because previous literature has suggested that self-reported pain may influence perceived self-efficacy but they are distinct constructs [26]. In contrast, larger correlation coefficients were anticipated because we assumed the results from a systematic review suggests that higher self-efficacy predicts higher performance as measured with FCE tests [27]. Our hypothesis about the expected correlation was also supported by two of our previous studies which compared the original SFS and FCE lifting tests [11, 13]. Furthermore, the authors expected that male patients would score higher on the M-SFS than age-matched females because males work more frequently in manual occupations, which usually entails higher average physical capacity [28].

**Table 1:** Four hypotheses for examining construct validity of the Modified Spinal Function Sort (M-SFS)

#n02	Reference test	The validity is not rejected if the strength of the relationship of M-SFS with	r cut-off values
1	Self-efficacy (SFS)	Self-reported perceived self-efficacy is large	0.50 ≤│r│
2	Pain disability (PDI)	Self-reported pain disability is medium	−0.30 ≤│r│ ≤ −0.49^a^
3	Pain (NRS)	Self-reported pain is medium	−0.30 ≤│r│ ≤ −0.49^a^
4	Lifting tests Lifting low Lifting high Horizontal lifting	Functional lifting tests is large	0.50 ≤│r│

^a^Negative correlation is expected because lower disability (Pain Disability Index and Oswestry Disability Index) or pain (Numeric Rating Scale) scores would correlate with higher function (M-SFS) scores. │r│ = Correlation coefficient, absolute value. The direction of the association depends on the scoring of the reference measure

The M-SFS was considered valid when the majority (≥80%) of the a priori hypotheses were not rejected [29]. A significance level of p < 0.05 was used. All analyses were performed using SPSS (Statistical Package for Social Sciences, Version 20, IBM Corp.).

## Results

### Participants

From December 2015 to June 2016, 62 subjects were included based on the inclusion criteria (Table 2).

**Table 2:** Characteristics of the patients (n = 62)

Characteristics, unit or scale	
Age (years)	38.5 (11.6)
Female, n (%)	21 (34.0)
Marital status, n (%)	
Married or co-habitation	16 (25.9)
Single	33 (53.2)
Divorced or living separated	11 (17.7)
Other (e.g. widowed)	2 (3.2)
Cultural background^e^, n (%)	
Swiss (-German)	48 (79.0)
Albanian	4 (6.5)
Turkish	3 (4.8)
Italian	2 (3.2)
Other	4 (6.5)
Duration of symptoms (days)*	304 (188.0; 571.5)
Affected area, primary, n (%)	
Low back	23 (37.1)
Neck	18 (29.0)
Leg	11 (17.7)
Arm	10 (16.1)
Work status: job contract, n (%)	240 (79.5)
Education^b^, n (%)	
Low	18 (29.0)
Intermediate	42 (67.7)
High	2 (3.3)
Physical work demands^c^, n (%)	
Sedentary (5 kg)	7 (11.3)
Light (5–10 kg)	8 (12.9)
Medium (10–25 kg)	15 (24.2)
Heavy (25–45 kg)	16 (25.8)
Very heavy (>45 kg)	16 (25.8)
FCE tests	
Lifting low (kg)	19.2 (7.8)
Lifting high (kg),^d^	11.75 (7.5; 15.0)
Lifting horizontally (kg),^e^	20.00 (18.1; 25.0)
Self-reported measures (scoring range)	
Functional ability (SFS, 0–200), SD	126.7 (44.42)
Pain disability (PDI, 0–70), SD	27.13 (8.52)
Pain now (NRS, 0–10), SD	4.02 (2.17)
Low back pain disability (ODI, 0–50), SD^f^	14.23 (6.3)

*If data have a skewed distribution median and an interquartile range (IQR), else mean and standard deviation are provided

^a^Mother language was used as a proxy for cultural background. Other = 1 Polish, 1 Tamil, 1 Portuguese, 1 Croatian

^b^Level of education: *low* no vocational training, primary or secondary school only, *intermediate* vocational training, *high* bachelor-, master or higher degree

^c^Physical work demands according to the Dictionary of occupational titles (DOT); *SFS* Spinal function sort, *PDI* Pain Disability Index, *NRS* Numeric Rating Scale, *ODI* Oswestry Disability Index

^d^Missing data for 12 participants

^e^Missing data for 6 participants

^f^Results from 23 participants with low back pain as the primary affected area

### Item Distribution and Internal Consistency

**Fig. 2:**
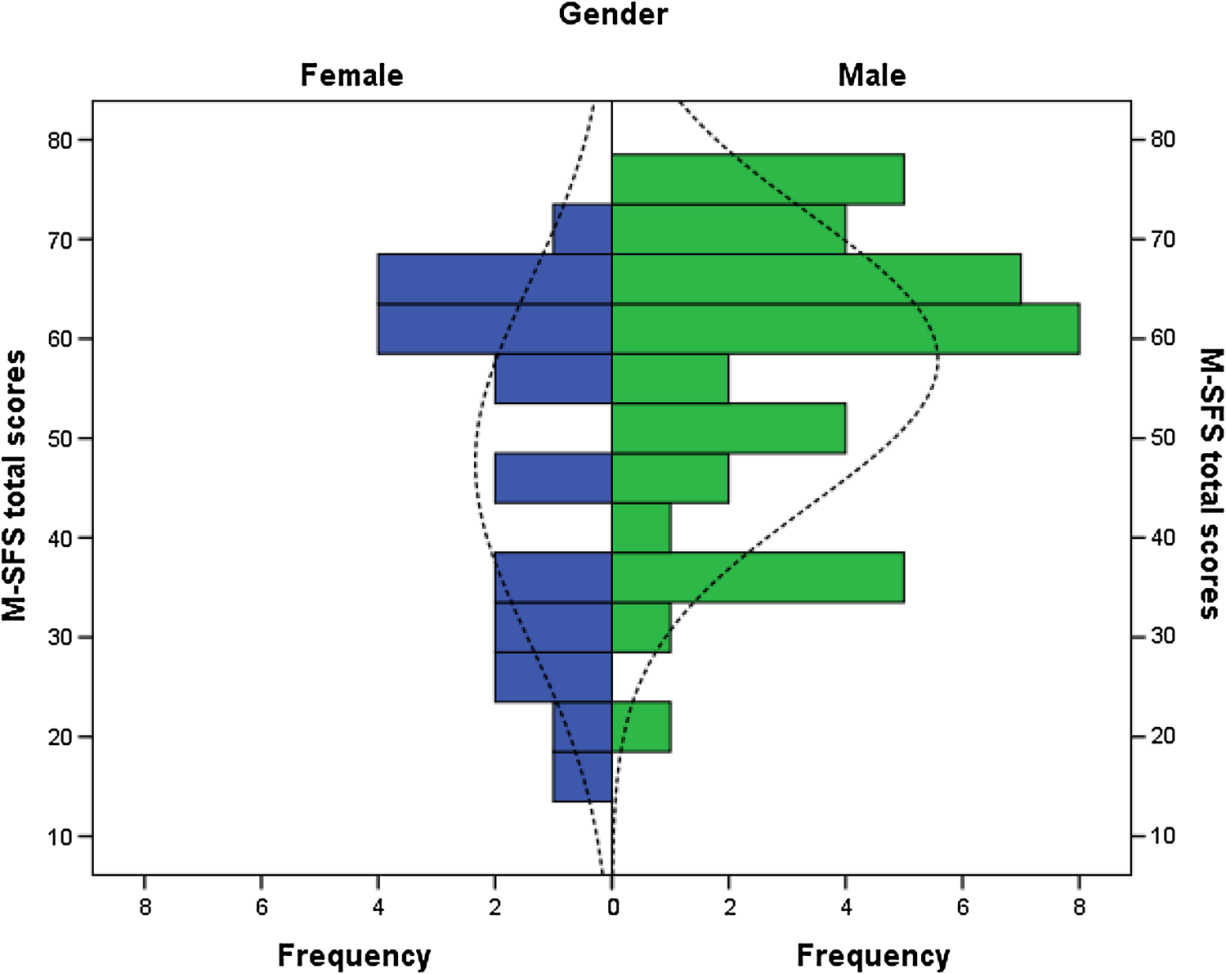
Distributions of the M-SFS total scores separated by gender

Item distribution showed no ceiling or floor effects. The M-SFS total score for all participants was 54.4 (SD 16.4) and 56.1 (SD 16.4) for test and retest, and ranged between 16 and 79, and 14 and 80 for test and retest, respectively. In the test, male participants scored 57.9 [median, Interquartile range (IQR) 49–69] and female 55.0 (median, IQR 30–64). In the retest, male participants scored 64.0 (median, IQR 52–72) and female 48.0 (median, IQR 28–64).Total score item distribution by gender of the first test is displayed in Fig. 2 (retest data available on request). Internal consistency was Cronbach’s alpha 0.94 and 0.95 for test and retest, respectively.

### Item-Structure Based on Principal Component Analysis (PCA)

PCA with fixed factors showed the presence of four components with initial Eigenvalues exceeding 1, explaining 50.2, 11.5, 6.2, and 5.7%, respectively, with a total of 74% of the variance. The inspection of the scree plot revealed four components. For the interpretation of the components Varimax rotation was executed. The rotated solution revealed the presence of a mixed structure of components showing distinct loadings >0.6 for 17 of 20 items, indicating reasonable evidence for multidimensionality (Table 3). Item 16 (walking) loaded on components 1, 2, 3, and 4 with values of 0.380, 0.398, 0.439 and −0.353, respectively.

**Table 3:** Factor loadings from principal component analysis of each M-SFS item

M-SFS item	Factor loadings (Varimax rotation)^a^			
	Factor 1	Factor 2	Factor 3	Factor 4
Place a box weighing 10 kg into the trunk of a car (Item 5)	0.903			
Place a box weighing 5 kg from a table to the floor (Item 14)	0.900			
Carry a bag weighing 5 kg in each hand (Item 7)	0.862			
Place a box weighing 10 kg from eye level to the floor (Item 11)	0.834			
Lift a box weighing 5 kg from the floor to eye level (Item 4)	0.826			
Take a 5 kg bag in each hand out of the trunk of a car (Item 10)	0.822			
Place a can weighing 2 ½ kg above your head (Item 1)	0.770			
Lift a 25 kg tool box from the floor to a bench (Item 18)	0.723			
Use a vacuum cleaner (pushing and pulling) (Item 8)	0.650			
Load or unload the dishwasher. (Item 19)	0.533			
Stand for a prolonged period of time in a forward leaning position (Item 15)		0.824		
Bending over frequently (Item 17)		0.818		
Prolonged sitting in a forward bent position (Item 6)		0.818		
Work for a prolonged period of time in a crouched or squatting position (Item 12)		0.696		
Wash dishes at a sink (Item 13)		0.500		
Work sitting down (Item 2)			0.738	
Take a bus (excess vibration) (Item 20)			0.725	
Remain standing for a prolonged period of time (Item 3)			0.705	
Walk for a prolonged period of time (Item 16)^b^				
Get into a car (Item 9)				0.777

^a^19 out of 20 items with factor loadings >0.6 are shown

^b^Item 16 (Walking) scored <0.5 (between 0.380 and 0.430) on all four components

### Test–Retest Reliability and Agreement

**Fig. 3:**
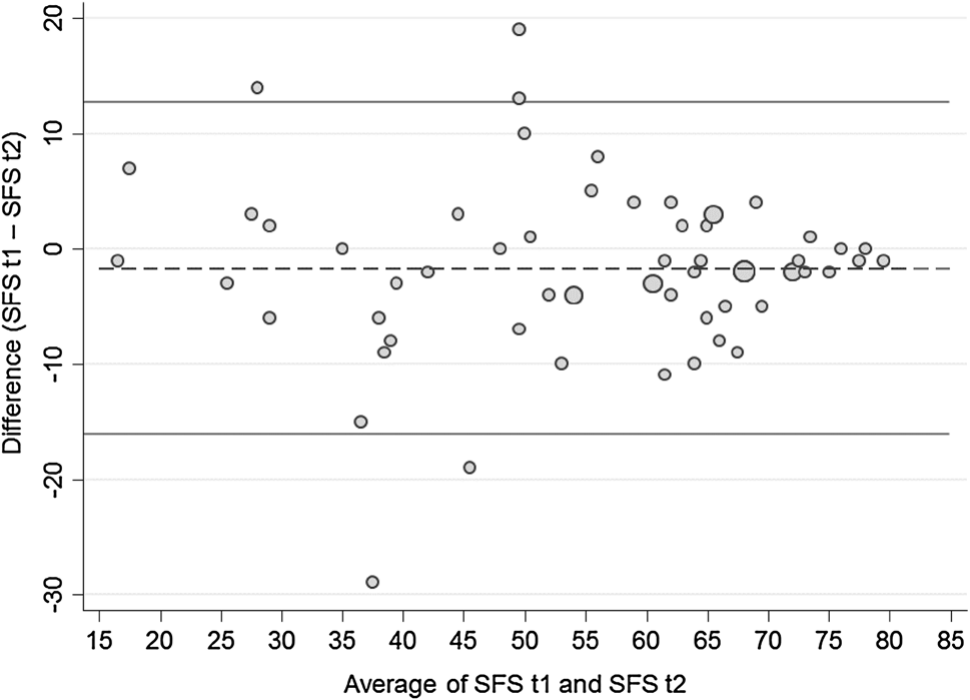
Bland–Altman plot of the M-SFS scores. The *middle line* represents the mean difference between the test and retest. The *lines above and below the middle line* represent the upper and lower limit of agreement, i.e. mean difference + 1.96 SD of the differences and mean difference −1.96 SD of the differences, respectively

The test–retest reliability measured with the ICC was 0.90 (95% CI 0.84–0.94). For the 62 patients in the reliability study, mean M-SFS scores for test and retest were 54.4 (mean, SD 16.4), and 56.1 (mean, SD 16.4), respectively. Mean difference in M-SFS score between test and retest was 1.7 (SD 7.3, *p* = .0.068). Hence LOA were +12.6 (upper limit) and −16.2 (lower limit) points (Fig. 3). Variances were related to the magnitude of the score.

### Construct Validation

Correlations for first test between the M-SFS and the original SFS were 0.89, and −0.49 with pain-related disability (PDI), and −0.37 and −0.33 with self-reported pain (NRS) for test and retest. Correlation coefficients between the M-SFS and FCE tests for the first test were 0.43, 0.46 and 0.52 for lifting low, lifting high and horizontal lifting, respectively. In patients with low back pain, correlation with self-reported disability (ODI) was −0.52. All correlations were significant (*p*-value <0.001). Correlations of M-SFS scores during retest were higher correlated with the FCE test-results compared to the first test, ranging from 0.50, 0.56 to 0.59 for lifting low, lifting high and lifting horizontally. Hence, the four hypotheses were not rejected and validity of M-SFS was confirmed.

## Discussion

This study assessed test–retest reliability and validity of the M-SFS in 62 patients with chronic nonspecific MSD in a work-related rehabilitation setting. Men rated their physical capacity on average higher than woman. No floor or ceiling effect of the items was observed. Internal consistency as well as test–retest reliability was high. Limits of agreement of 14.8 suggest that true changes in individuals are above 18.1 of the total score. We found a high correlation with the original SFS [11], suggesting a high criterion validity of the newly developed M-SFS. Low correlations with pain ratings and moderate correlations with self-reported pain disability as well as performed physical capacity suggest acceptable construct validity. None of the a priori defined hypotheses (see Table 1) were rejected and validity of M-SFS for this setting was confirmed. Principal component analysis confirmed the existence of main components *lifting & carrying, activities with bending of the spine, prolonged body postur*es. These components are in line with the themes that emerged from the previous mixed-method study based on expert opinions, interviews with patients and item analysis from clinical study-data [14].

The lower correlations of the M-SFS with physical performance than previously found of the original SFS can, from our point of view, be explained by several factors. First the time of measurement, which in the current study was at the beginning of the rehabilitation stay while the original SFS was compared with physical performance at the end of rehabilitation. It has formerly been shown that self-reported physical capacity becomes more accurate with repeated testing, which happened in the retest [11]. Second, the items of the M-SFS focus less on lifting than the original SFS and include new items with postural activities and ambulation tasks. Third, several items depicting heavy-lifting tasks contained in the original SFS were removed, and finally, as reported in other studies, self-reported and performance-based measures of activity are related but distinct [28, 29].

This study revealed on average a 1.7 higher rated physical capacity at the retest measurement. The highest contribution to this systematic error in measurement results from patients with lower M-SFS scores. These patients showed higher total score variations from test to retest (see also Fig. 3). Obviously, such variation results in increased LoA. Large LoA scores in patient-reported outcome measure are common in pain patients [7, 30, 31]. We are unaware of accepted guidelines for cutoff points of LoA [30]. The observed 18% measurement error ratio (in our study is markedly below that score). In addition, sample characteristics need to be taken into account when interpreting the results. Previous studies reported that the LoA values of the original SFS administered by injured workers from a French-speaking rehabilitation setting were ±11 (6% of the maximal score of 200) while in the German-speaking area the values were ±27 (14% of the maximal score). Another study reported LoA values of ±33 (16% of the maximal score) of the original SFS in workers with subacute whiplash injuries [7]. The authors argued that beside individual differences, the cultural and legal context may also influence test–retest values [6, 7].

### Practical Implications of Measuring PSE

Guiding rehabilitation of workers with MSD by using the M-SFS may supported for the following reasons: First, the M-SFS attempts to measure PSE with specific, picture-based questions. It has been suggested that the closer a question to actual behavior, the more accurately behavior was predicted [32]. Therefore, the M-SFS may complement direct observations and information from peers to help guide treatment and RTW interventions. Second, the M-SFS assists the clinician to maintain the focus on function throughout the treatment by identifying and intervene on specific items with low scores. Third, substantial discrepancies between the self-reported ability measured with the M-SFS and observed ability may indicate psychological problems which may require more exploration [33]. Fourth, there is a need for measures which take into account culturally diverse patient groups [34, 35]. Hence, the M-SFS lends to translation into other languages because the short text is supplemented by pictorial illustrations [36].

### Limitations

Besides its strengths, this study has limitations. First, additional studies are needed to compare the M-SFS to other work-related measures including tasks of postural tolerance, ambulation or repetitive bending of the spine. Second, the missing follow-up after rehabilitation prevents assessment of the predictive validity of the M-SFS for return to work. A cutoff score of <100 of the original SFS has shown to be predictive for non-return to work and was achieved by 42% of the responding patients with nonspecific chronic low back pain [11]. The equivalent M-SFS score at the 42nd percentile is <56. We therefore hypothesize that an M-SFS score <56 will be predictive for non-return to work in patients with MSD and recommend its cautious use until further research determines the predictive validity of the M-SFS. Third, the small sample size, which may alter results of the PCA. However, it has been suggested that if factor loadings are above 0.6, even relatively small samples (<100) may be perfectly adequate [37]. In this study 17 out of 20 items had factor loadings >0.6. Nevertheless, the factor structure of the M-SFS should be studied with larger samples. Further research on the M-SFS should also address the anticipated effect of repeated testing, investigate the ability to measure change (responsiveness) and predictive validity for return to work. Finally, transcultural adaptation and translation of the German M-SFS into other languages as well as validation in other settings and populations is needed.

## Conclusion

Perceived self-efficacy for work-related tasks can reliably and validly be assessed with the M-SFS. Measurement properties of the M-SFS were similar to those of the original SFS while time for administration is substantially reduced. Hence, we recommend the use of the modified-version of the SFS. Further research should investigate the properties of the M-SFS with regards to other work-related measures and the proposed M-SFS score of <56 for its predictive validity for non-RTW.
